# Structural Analysis of *Saccharomyces cerevisiae* Dihydroorotase Reveals Molecular Insights into the Tetramerization Mechanism

**DOI:** 10.3390/molecules26237249

**Published:** 2021-11-29

**Authors:** Hong-Hsiang Guan, Yen-Hua Huang, En-Shyh Lin, Chun-Jung Chen, Cheng-Yang Huang

**Affiliations:** 1 Life Science Group, Scientific Research Division, National Synchrotron Radiation Research Center, Hsinchu 33076, Taiwan; d938245@oz.nthu.edu.tw; 2 School of Biomedical Sciences, Chung Shan Medical University, No.110, Sec.1, Chien-Kuo N. Rd., Taichung City 402, Taiwan; cicilovev6@gmail.com; 3 Department of Beauty Science, National Taichung University of Science and Technology, No.193, Sec.1, San-Min Rd., Taichung City 403, Taiwan; eslin7620@gmail.com; 4 Department of Biotechnology and Bioindustry Sciences, National Cheng Kung University, Tainan City 701, Taiwan; 5 Department of Physics, National Tsing Hua University, Hsinchu 30043, Taiwan; 6 Department of Biological Science and Technology, National Chiao Tung University, Hsinchu 300193, Taiwan; 7 Department of Medical Research, Chung Shan Medical University Hospital, No.110, Sec.1, Chien-Kuo N. Rd., Taichung City 402, Taiwan

**Keywords:** dihydroorotase, tetramerization, pyrimidine biosynthesis, CAD, dihydropyrimidinase, allantoinase, thermostability

## Abstract

Dihydroorotase (DHOase), a dimetalloenzyme containing a carbamylated lysine within the active site, is a member of the cyclic amidohydrolase family, which also includes allantoinase (ALLase), dihydropyrimidinase (DHPase), hydantoinase, and imidase. Unlike most known cyclic amidohydrolases, which are tetrameric, DHOase exists as a monomer or dimer. Here, we report and analyze two crystal structures of the eukaryotic *Saccharomyces cerevisiae* DHOase (ScDHOase) complexed with malate. The structures of different DHOases were also compared. An asymmetric unit of these crystals contained four crystallographically independent ScDHOase monomers. ScDHOase shares structural similarity with *Escherichia coli* DHOase (EcDHOase). Unlike EcDHOase, ScDHOase can form tetramers, both in the crystalline state and in solution. In addition, the subunit-interacting residues of ScDHOase for dimerization and tetramerization are significantly different from those of other DHOases. The tetramerization pattern of ScDHOase is also different from those of DHPase and ALLase. Based on sequence analysis and structural evidence, we identify two unique helices (α6 and α10) and a loop (loop 7) for tetramerization, and discuss why the residues for tetramerization in ScDHOase are not necessarily conserved among DHOases.

## 1. Introduction

**Figure 1:**
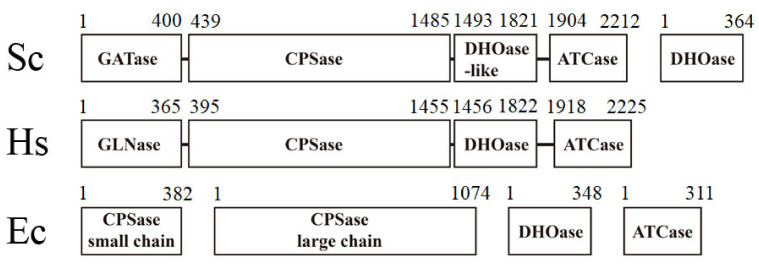
The gene products for the first three reactions of pyrimidine biosynthesis are different among species. The higher eukaryotic human CAD consists of DHOase, CPSase, and ATCase domains fused covalently. Sc, *Saccharomyces cerevisiae*; Ec, *Escherichia coli*. Bacterial DHOase, CPSase, and ATCase function separately. However, CPSase and ATCase activities in *S. cerevisiae* are present in a single bifunctional protein, Ura2. Ura2 is a CAD-like polypeptide that contains a defective DHOase-like domain. Yeasts have a monofunctional DHOase, encoded by an independent gene, which is the protein that is crystallized in this study.

Dihydroorotase (DHOase) catalyzes the reversible cyclization of *N*-carbamoyl aspartate (CA-asp) to dihydroorotate (DHO) for the biosynthesis of pyrimidine nucleotides [1]. Although the de novo synthesis process of pyrimidines is conserved among all species, the gene products responsible for these enzyme activities differ (Figure 1). In mammals, DHOase is a part of a single trifunctional polypeptide of 240 kDa, namely, carbamoyl phosphate synthetase (CPSase)/aspartate transcarbamoylase (ATCase)/DHOase protein (CAD), that it self-assembles into a hexamer of 1.5 MDa [2]. In yeasts, CPSase and ATCase are present in a single bifunctional protein Ura2, which is a CAD-like polypeptide that contains a defective DHOase-like domain [3]. In most prokaryotic organisms, CPSase, ATCase, and DHOase are expressed separately and function independently [4]. *Aquifex aeolicus* DHOase (AaDHOase) is active only when complexed with AaATCase [5]. Given that CAD is a key enzyme for the cell’s progression through the S phase of the cell cycle and controls the cell proliferation in mammalian cells [6,7,8], these distinct differences among species may indicate that DHOase is a promising target [9,10,11] for potential antimalarial, anticancer, and antipathogen chemotherapy [12,13].

On the basis of known amino acid sequences and phylogenetic analyses, two major groups of DHOases are classified [14]. These two types of DHOases share a low level of protein sequence identity (less than 20%). AaDHOase and *Bacillus anthracis* DHOase (BaDHOase) are type I DHOases. Counterparts from eubacteria, fungi, and plants are type II DHOases. Recently, the DHOase domain of human CAD (huDHOase) was reclassified as the type III DHOase, due to unique structural features [15]. All known DHOases can exist as a monomer or dimer. In the dimeric structure of *Escherichia coli* DHOase (EcDHOase) [16], each subunit folds into a TIM barrel motif with eight strands of parallel β-sheet flanked on the outer surface by α-helices. The complex structures of EcDHOase [17], BaDHOase [18], and huDHOase [15,19] reveal that despite the evolutionary divergence, an important flexible loop [17] as a lid within the active site of DHOase for catalysis and substrate binding is conserved from *E. coli* to humans. This flexible loop extends toward the active site when CA-asp is bound (loop-in mode) or moves away from the active site, facilitating the product DHO release (loop-out mode).

**Figure 2:**
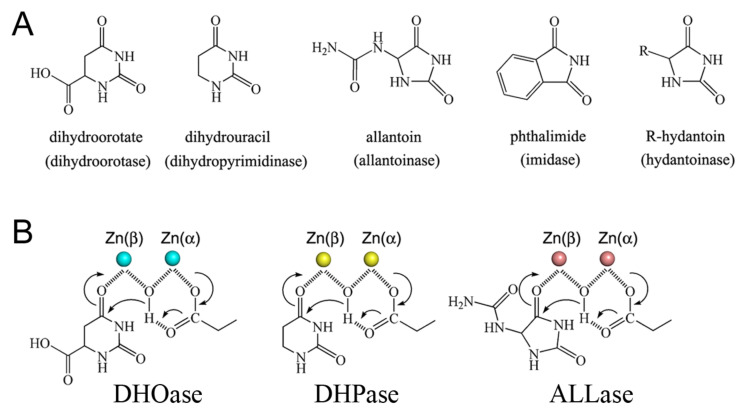
Properties of the cyclic amidohydrolase family. (**A**) Substrate of dihydroorotase, dihydropyrimidinase, allantoinase, imidase, and hydantoinase. Dihydroorotase cannot use the substrates of other cyclic amidohydrolases as substrate, despite having a similar active site. (**B**) The chemical mechanisms of dihydroorotase, dihydropyrimidinase, and allantoinase. The hydrolysis of the substrates likely undergoes three steps: the hydrolytic water molecule must be activated for nucleophilic attack, the amide bond of the substrate must be made more electrophilic by polarization of the carbonyl-oxygen bond, and then the leaving-group nitrogen must be protonated as the carbon nitrogen bond is cleaved. The metal ions are shown as circles.

On the basis of an analysis of the amino acid sequences, DHOase [20] was suggested to be a member of the cyclic amidohydrolase family [21,22], which also includes dihydropyrimidinase (DHPase) [23,24,25,26,27], allantoinase (ALLase) [28,29,30], hydantoinase (HYDase) [31,32], and imidase [33,34,35]. These metal-dependent enzymes catalyze the hydrolysis of the cyclic amide bond of each substrate in either 5- or 6-membered rings in the metabolism of purines and pyrimidines (Figure 2A). Almost all enzymes contain the binuclear metal center that consists of four His, one Asp, and one post-translational carbamylated Lys (Kcx) residue. Although these cyclic amidohydrolases may use a similar active site and mechanism for catalysis (Figure 2B), no substrate overlapping is observed for DHOase, DHPase, and ALLase [36]. The post-carbamylated Lys is needed for the enzyme activity and the self-assembly of the binuclear metal center [28,31,37]. Despite having a similar active site, different metal dependences are found. *Tetraodon nigroviridis* DHPase with one Zn ion is the active form for catalysis [26]. Only one Zn ion is found in the active site of AaDHOase [38]. In huDHOase, a novel third Zn ion bound to a rare histidinate ion at the active site [15] is functionally important, but is not found in any DHOase. Thus, structural analyses are still needed to decipher the architecture and the function of different DHOases.

DHOases may have distinct situations for oligomer evolution. The dimerization mode of EcDHOase is significantly different from that of huDHOase [15]. Because fungi Ura2 (CPSase–ATCase fused with DHOase-like domain) owns its DHOase-like domain for activity [3,39,40], fungi DHOase may not require the formation of multicomplexes with Ura2. The fungi DHOase may be no longer needed to coevolve with the CPSase and the ATCase (Figure 1). Thus, analyzing the structural differences in fungi DHOase is of considerable interest. Very recently, the crystal structure of *Saccharomyces cerevisiae* DHOase (ScDHOase) has been reported [41,42,43]; however, the oligomeric state of this enzyme has not been analyzed yet.

In this study, we found that ScDHOase could form dimers and tetramers both in the crystalline state and in solution. ScDHOase exhibited higher thermostability than huDHOase. Based on sequence analysis and structural evidence, we identified unique tetramerization helices and the loop in ScDHOase. Structural comparison indicated that ScDHOase used different mechanisms for forming oligomers among DHOases and other cyclic amidohydrolases, which may be due to the evolutionary diversity.

## 2. Materials and Methods

### 2.1. Protein Expression and Purification

The construction of huDHOase and ScDHOase expression plasmids were reported previously [44]. The recombinant protein was purified using the protocol described previously [44]. Briefly, *E. coli* BL21(DE3) cells were transformed with the expression vector, and the overexpression of the expression plasmids was induced by incubating with 1 mM isopropyl thiogalactopyranoside. The protein was purified from the soluble supernatant by using the Ni^2+^-affinity chromatography (HiTrap HP; GE Healthcare Bio-Sciences), eluted with Buffer A (20 mM Tris–HCl, 250 mM imidazole, and 0.5 M NaCl, pH 7.9), and dialyzed against a dialysis buffer (20 mM Tris–HCl and 0.1 M NaCl, pH 7.9). The protein purity remained at >97%, as determined using SDS–PAGE (Mini-PROTEAN Tetra System; Bio-Rad, Hercules, CA, USA).

### 2.2. Crystallization Experiments

Before crystallization, the purified ScDHOase was concentrated to 11 mg/mL. Crystallization trials were performed using the hanging drop vapor diffusion method through a liquid-handling robot (Mosquito, TTP Labtech) for high-throughput screening. The crystals of ScDHOase were grown at room temperature through the hanging drop vapor diffusion in 16% PEG 4000 and 100 mM imidazole–malate, pH 7.0. The crystals of ScDHOase were also grown in 19% PEG 4000 and 100 mM imidazole–malate, pH 7.5. These conditions were further optimized to improve the diffraction quality and resolution. The crystals reached full size in 9–12 days. The crystals were transferred from a crystallization drop into the cryoprotectant solution (2 μL) with precipitant solution containing glycerol (25–30%) for a few seconds, mounted on a synthetic nylon loop (0.1–0.2 mm, Hampton Research), and flash cooled in liquid N_2_. The crystals of ScDHOase were validated in the beamline 15A of the National Synchrotron Radiation Research Center (NSRRC; Hsinchu, Taiwan) and in the beamline BL44XU at SPring-8 (Harima, Japan).

### 2.3. X-ray Diffraction Data and Structure Determination

The native and the Zn-anomalous data were collected at beamline BL44XU at SPring-8 (Harima, Japan), with MX300–HE CCD detector, and at beamline 15A at the NSRRC (Hsinchu, Taiwan), with MX300–HS CCD detector. Data sets were indexed, integrated, and scaled by HKL-2000 [45] and XDS [46]. The Zn–SAD data were collected at the wavelength of 1.2819 Å by using the best crystal of the ScDHOase–malate complex. The initial phase, density modification, and model building were performed using the AutoSol program [47] in the PHENIX. The iterative model building and the structure refinement were performed using Refmac in the CCP4 software suite [48] and Phenix.refine in the PHENIX software suite [49]. The correctness of the stereochemistry of the models was verified using MolProbity [50]. All refinement statistics are summarized in Table 1. The atomic coordinates and the related structure factors of ScDHOases were deposited in the PDB with accession codes 6L0A (malate, pH 7) and 6L0J (malate, pH 7.5).

### 2.4. Enzyme Assay

A rapid spectrophotometric assay was used to determine the activity of DHOase [28,36]. Briefly, the hydrolysis of DHO was measured at 25 °C as the decrease in absorbance at 230 nm. The purified DHOase was added to a 2 mL solution containing 0.5 mM DHO and 100 mM Tris–HCl at pH 8.0 to start the reaction. The extinction coefficient of DHO was 0.92 mM^−1^·cm^−1^ at 230 nm. The hydrolysis of DHO was monitored using a UV/vis spectrophotometer (Hitachi U 3300; Hitachi High-Technologies, Tokyo, Japan). A unit of activity was defined as the amount of enzyme catalyzing the hydrolysis of 1 μmol DHO/min, and the specific activity was expressed in terms of units of activity per mg of enzyme.

### 2.5. Chemical Crosslinking

The oligomerization state of DHOase was analyzed through the chemical crosslinking by using glutaraldehyde [51], as described previously for DnaT [52] and SsbC [53]. DHOase (3 μM) was incubated with increasing concentration of glutaraldehyde (0.5%) at 4 °C for 20 min. The reactions were stopped by adding the SDS sample buffer, and fractionated using the Coomassie Blue-stained SDS–PAGE.

### 2.6. Gel Filtration Chromatography

Gel filtration chromatography was carried out by the AKTA-FPLC system (GE Healthcare Bio-Sciences, Piscataway, NJ, USA). In brief, purified protein (10 mg/mL) in Buffer B (20 mM MES and 100 mM NaCl, pH 6.0) was applied to a Superdex 200 prep grade column (GE Healthcare Bio-Sciences, Piscataway, NJ, USA) equilibrated with the same buffer. This column was operated at a flow rate of 0.5 mL/min, and the proteins were detected at 280 nm. The column was calibrated with proteins of known molecular weight: thyroglobulin (670 kDa), γ-globulin (158 kDa), ovalbumin (44 kDa), myoglobin (17 kDa), and vitamin B_12_ (1.35 kDa).

## 3. Results and Discussion

### 3.1. Crystals of ScDHOase Contained Four Monomers per Asymmetric Unit

The DHOase activity was found in all organisms for the biosynthesis of pyrimidine nucleotides, but phylogenetic and structural analyses revealed at least three different DHOase forms [1,15]. As a eukaryotic DHOase, ScDHOase may be an evolutionary link (Figure 1) between the bacterial DHOase and the higher eukaryotic DHOase domain of CAD. Very recently, we have reported the crystal structures of ScDHOase in complex with 5-fluorouracil [43], 5-aminouracil [43], plumbagin [42], and 5-fluoroorotate [41]. Currently, we noticed that all these crystals contained four monomers per asymmetric unit. Is it a mere coincidence? Given that all known DHOases exist as a monomer or dimer, we attempted to investigate whether ScDHOase can form tetramers. Initially, we analyzed the malate-complexed crystal structures of ScDHOase solved previously at pH 6.0 (PDB entry 6L0G), 6.5 (PDB entry 6L0I), 7.0 (PDB entry 6L0H), and 9.0 (PDB entry 6L0K) [43]. The resolutions of these structures ranged from 2.1 to 3.3 Å [43]. In this study, we also crystallized and determined two new structures of ScDHOase at a resolution of 1.79 (PDB entry 6L0A) and 1.93 Å (PDB entry 6L0J) for comparison (Table 1). Although these two crystals of ScDHOase also contained four monomers per asymmetric unit, their dimer–dimer interaction modes were different.

### 3.2. Overall Structure and Tetramer Formation of the Complex Form I (PDB Entry 6L0A)

**Figure 3:**
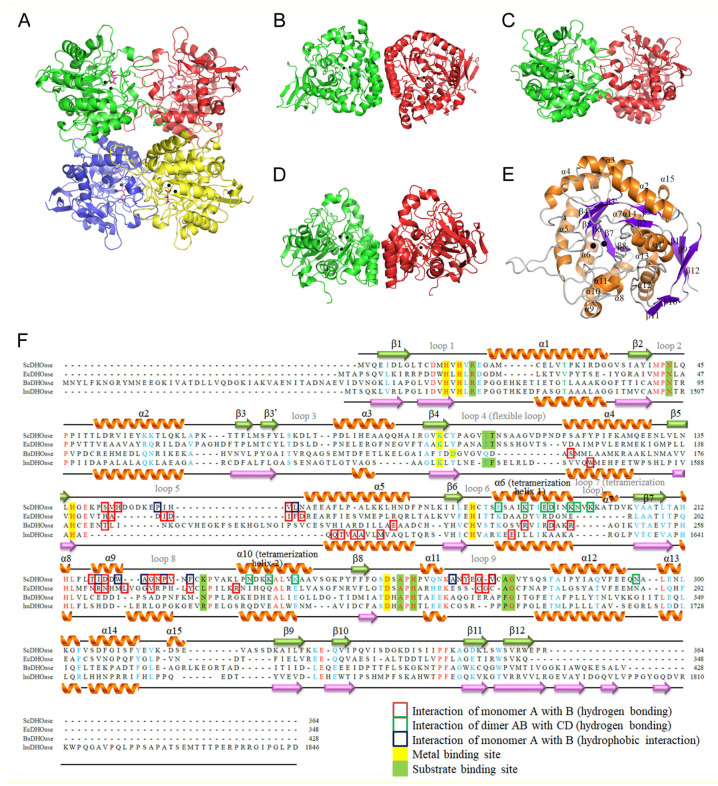
Structure of DHOases. (**A**) Ribbon diagram of an ScDHOase tetramer (PDB entry 6L0A). Each ScDHOase monomer is color-coded. Two zinc ions in the active site are presented as black spheres. Malate (light magenta) was found in the active site of each subunit. (**B**) Structure of the type I enzyme BaDHOase. (**C**) Structure of the type II enzyme EcDHOase. (**D**) Structure of the type III enzyme huDHOase. (**E**) Ribbon diagram of an ScDHOase monomer with the secondary structures labeled. The global architecture of each ScDHOase monomer (PDB entry 6L0A) revealed a TIM barrel structure and consists of 15 α-helices, 12 β-sheets, and two zinc ions. (**F**) Structure-based sequence alignment of ScDHOase, EcDHOase, BaDHOase, and huDHOase. The labeled secondary structural elements of ScDHOase and huDHOase are shown above and below the alignment, respectively. The amino acids that are involved in monomer–monomer and dimer–dimer interface via hydrogen bonding are boxed in red and green, respectively. The amino acids that are involved in monomer–monomer interface via hydrophobic interactions are boxed in dark blue. Amino acid residues displaying 100% identity are colored in red, and those displaying similarity are colored in cyan. The metal and substrate binding sites are shaded in yellow and green.

The complex form I was obtained in 16% PEG 4000 and 100 mM imidazole–malate, pH 7.0. We could not obtain any crystal of the apoenzyme. The removal of malate from the reservoirs resulted in no crystal appearance. The crystals of ScDHOase belonged to space group P2_1_ with four molecules per asymmetric unit (Figure 3A). Structurally, the oligomerization mode of ScDHOase was significantly different from that of the type I enzyme BaDHOase (Figure 3B), the type II enzyme EcDHOase (Figure 3C), and the type III enzyme huDHOase (Figure 3D). The global architecture of each ScDHOase monomer revealed a TIM barrel structure and consisted of 15 α-helices, 12 β-sheets, and 2 Zn ions (Figure 3E). The active site of ScDHOase containing four His (i.e., H14, H16, H137, and H180), one Asp (i.e., D258), and one carbamylated Lys (i.e., Kcx98), which were required for the metal binding [16], was similar to that of EcDHOase and other members of the cyclic amidohydrolase family, such as ALLase and DHPase. Arg18, Asn43, and His262 in ScDHOase (Figure 3F) structurally corresponded to Arg20, Asn44, and His254 in EcDHOase, which were the sites for the binding of DHO via electrostatic interactions [54]. The two Thr residues, Thr105 and Thr106 [17], important for stabilizing the transition state, were also conserved in ScDHOase (Figure 3F). Given that these two type II enzymes possessed a similar active site, ScDHOase and EcDHOase might use a similar mechanism for the catalysis. However, the oligomerization mechanisms between ScDHOase and EcDHOase were different (see below).

### 3.3. Overall Structure and Tetramer Formation of the Complex Form II (PDB Entry 6L0J)

**Figure 4:**
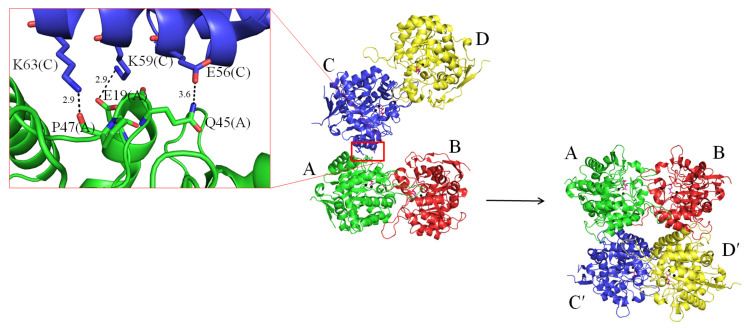
Structure of the complex form II. An asymmetric unit contains four crystallographically independent ScDHOase monomers D-C-A-B. Since the two dimers of ScDHOase associated via few contacts (E56(C)–Q45(A), K59(C)–E19(A), and K63(C)–P47(A)) to create the tetramer, it was thought that the tetrameric state may be possibly due to crystal packing forces. Crystallographically related tetramer A-B-C′-D′ was formed and further stabilized via many hydrogen bonds and salt bridges. This tetramerization mode was similar to that of the complex form I.

The complex form II was obtained in 19% PEG 4000 and 100 mM imidazole–malate, pH 7.5. In this packing mode, the four molecules formed two pairs of dimers, C-D and A-B, respectively (Figure 4). Since the two dimers of ScDHOase associated via few contacts (E56(C)–Q45(A), K59(C)–E19(A), and K63(C)–P47(A)) to create the tetramer, it was thought that the tetrameric state may be possibly due to crystal packing forces. We noted that in this crystal, another crystallographically related tetramer A-B-C′-D′ was formed and further stabilized via many hydrogen bonds and salt bridges. This tetramerization mode was similar to that of the complex form I.

### 3.4. Oligomeric State of ScDHOase in Solution

**Figure 5:**
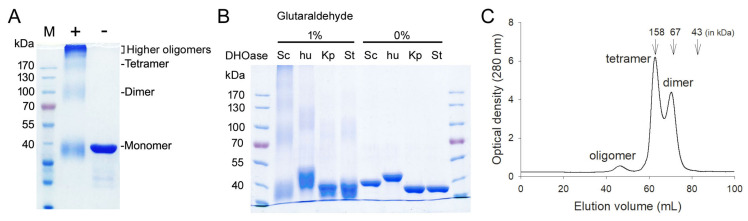
Oligomeric state of ScDHOase in solution. (**A**) Glutaraldehyde crosslinking of ScDHOase. ScDHOase (3 μM) was incubated with glutaraldehyde at 4 °C for 20 min. Coomassie Blue-stained SDS-PAGE of the resulting samples and molecular mass standards are shown. At 0.5% glutaraldehyde, the monomer of ScDHOase decreased evidently and was covalently crosslinked to different oligomers. The monomeric (~41 kDa), dimeric (~83 kDa), tetrameric (~170 kDa), and the higher oligomeric forms of ScDHOase were observed. (**B**) Glutaraldehyde crosslinking of different DHOases. DHOase (3 μM) was incubated with glutaraldehyde (1%) at 4 °C for 20 min. huDHOase, KpDHOase, and StDHOase did not form any tetramer, and only bands corresponding with the monomeric and the dimeric forms of huDHOase, KpDHOase, and StDHOase were found. In this condition, the number of ScDHOase monomers significantly decreased more than those of other DHOases through the crosslinking reagent. (**C**) Gel filtration chromatographic analysis. Gel filtration chromatography was carried out by the AKTA-FPLC system. The corresponding peaks show the eluting ScDHOase. The column was calibrated with proteins of known molecular weight: thyroglobulin (670 kDa), γ-globulin (158 kDa), ovalbumin (44 kDa), myoglobin (17 kDa), and vitamin B12 (1.35 kDa). The results revealed that two species of ScDHOase with elution volume of 62.83 and 70.47 mL did coexist in solution.

All known DHOases can exist as a monomer or dimer. However, an asymmetric unit of the ScDHOase crystals always contained four crystallographically independent monomers. Our ScDHOase structure showed four highly associated ScDHOase molecules (Figure 3A), implying a tetramer forming in the crystalline state. We performed the chemical crosslinking of ScDHOase by using glutaraldehyde to further substantiate the observation from the crystal structure (Figure 4). Glutaraldehyde is one of the most effective protein crosslinking reagents via reacting with amine groups in protein [51]. At 0.5% glutaraldehyde, the monomer of ScDHOase decreased evidently, and was covalently crosslinked to different oligomers. The monomeric (~41 kDa), dimeric (~83 kDa), tetrameric (~170 kDa), and the higher oligomeric forms of ScDHOase were observed clearly (Figure 5A). For comparison, purified huDHOase [42], *Klebsiella pneumonia* DHOase (KpDHOase) [28], and *Salmonella enterica* serovar Typhimurium LT2 DHOase (StDHOase) [44] were also used for this analysis (Figure 5B). The molecular mass of these purified DHOases followed the order: huDHOase > ScDHOase > KpDHOase and StDHOase (Figure 5B). The individual DHOase (3 μM) was incubated with 1% glutaraldehyde at 4 °C for 20 min. By contrast, huDHOase, KpDHOase, and StDHOase did not form any tetramer, and only bands corresponding with the monomeric and the dimeric forms of huDHOase, KpDHOase, and StDHOase were found. In this condition, the number of ScDHOase monomers significantly decreased more than those of other DHOases through the crosslinking reagent (Figure 5B), suggesting the oligomerization nature of ScDHOase. Consequently, the glutaraldehyde crosslinking result showed that ScDHOase could occur as a tetramer in solution, and this finding was consistent with the result revealed by the crystal structure (Figure 3A).

The gel filtration chromatography was also conducted for analyzing the oligomerization stage of ScDHOase (Figure 5C). The results revealed that two species of ScDHOase with elution volume of 62.83 and 70.47 mL did coexist. Calculated from the standard linear regression equation, the native molecular masses of ScDHOase were estimated to be 164 and 79 kDa, respectively. The native molecular masses of ScDHOase were approximately 4.1 and 2.0 times the molecular mass of an ScDHOase monomer (40 kDa), respectively. Thus, ScDHOase could form dimers and tetramers in solution.

The different oligomeric states for these DHOases may be linked to the evolutionary diversity. The higher eukaryotic human CAD consists of DHOase, CPSase, and ATCase domains that fuse covalently (Figure 1). Bacterial DHOase, CPSase, and ATCase function separately. Thus, three genes for bacteria and only one gene for humans are needed and regulated, respectively, in the first three reactions of the de novo pyrimidine synthesis. However, the CPSase and the ATCase activities in *S. cerevisiae* are present in a single bifunctional protein Ura2 [40]. We speculate that ScDHOase may not require the formation of multicomplexes with ScUra2 during evolution. Thus, similar to the thermostable tetrameric DHPase, HYDase, and ALLase, ScDHOase may gradually evolve itself toward forming a tetramer for stability via the convergent evolution [21,55]. Whether ScDHOase needs to function as a tetramer or higher oligomer in association with ScUra2 (CPSase–ATCase) in *S. cerevisiae* remains uncertain because ScUra2 owns its DHOase-like domain for activity. The crystal structure of Ura2 is highly desired for this investigation.

### 3.5. Thermostability of ScDHOase

ScDHOase could form dimers and tetramers in solution (Figure 5). We performed indirect thermostability experiments to analyze the stability of ScDHOase (Table 2). The activity of ScDHOase incubated at 40 °C, 50 °C, 60 °C, and 70 °C for 10 min decreased by 3%, 21%, 48%, and 91%, respectively. huDHOase was also analyzed. Under the same conditions, the activity of huDHOase decreased more significantly. Thus, ScDHOase exhibited higher thermostability than huDHOase.

### 3.6. Tetramerization Mode of ScDHOase

**Figure 6:**
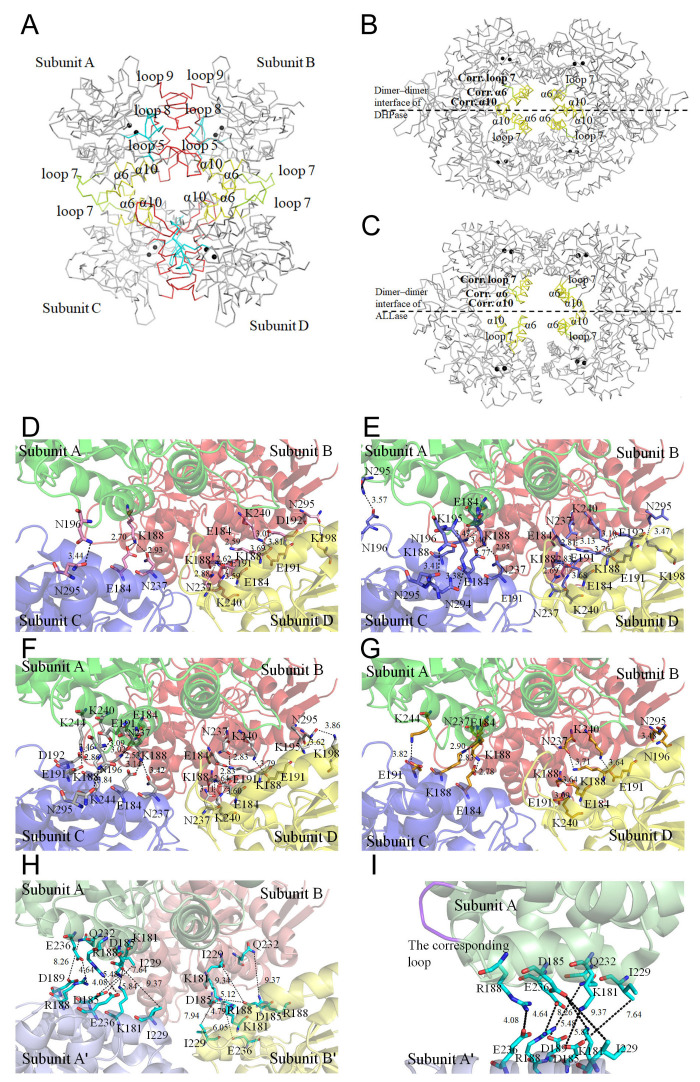
Tetramerization mechanism. (**A**) The Cα trace of an ScDHOase tetramer (PDB entry 6L0H). The tetramerization helices (α6 and α10) and loop (loop 7) are colored in yellow and limon, respectively. The dimerization loops 5, 8, and 9 are colored in red. The catalytic loop is colored in cyan. Two zinc ions in the active site are presented as black spheres. (**B**) The Cα trace of a *Thermus* sp. DHPase tetramer (PDB entry 1GKQ). (**C**) The Cα trace of an EcALLase tetramer (PDB entry 3E74). The helices and loop corresponding to the tetramerization helices 1, 2, and loop of ScDHOase are also involved in the tetramer formation of DHPase and ALLase, but their interaction modes are different. (**D**) The formation of hydrogen bonds at the dimer AB–dimer CD interface of ScDHOase at pH 6. The distance (Å) of the residues is shown. (**E**) The formation of hydrogen bonds at the dimer AB–dimer CD interface of ScDHOase at pH 6.5. (**F**) The formation of hydrogen bonds at the dimer AB–dimer CD interface of ScDHOase at pH 7. (**G**) The formation of hydrogen bonds at the dimer AB–dimer CD interface of ScDHOase at pH 9. (**H**) The corresponding residues at the crystallographically related dimer AB–dimer A’B’ interface of EcDHOase. The distance of these corresponding residues in EcDHOase was too far to interact with each other. (**I**) The corresponding loop at the crystallographically related monomer A–monomer A’ interface of EcDHOase. The loop (purple) in EcDHOase corresponding to the tetramerization loop of ScDHOase was too short to interconnect.

In this study, we identified that ScDHOase could form dimers and tetramers in solution (Figure 5). The crystal structure reveals that the α6 (yellow), α10 (yellow), and loop 7 (limon) in the individual subunit contributed to the tetramer formation of ScDHOase (Figure 6A). We designated α6, α10, and loop 7 as the tetramerization helix 1, tetramerization helix 2, and tetramerization loop, respectively (Figure 3F). The helices and loop corresponding to the tetramerization helices 1 and 2 and the loop of ScDHOase in DHPase (Figure 6B) and ALLase (Figure 6C) were also involved in their tetrameric formation, but the overall patterns were different. Many pH-dependent H bonds at the dimer AB–dimer CD interface of ScDHOase were formed (Table 3 and Figure 6D–G). We also compared the structure of this enzyme with that of EcDHOase (PDB entry 2EG6) to assess why the ScDHOase could form a tetramer. The dimer–dimer interface of these enzymes differed in terms of the residue composition in the tetramerization helices 1 and 2 and the tetramerization loop (Figure 3F) and the length of the tetramerization loop. Although these enzymes were originally classified in the type II DHOase, the important residues located at the dimer–dimer interface for the tetramer formation were almost different (Figure 3F and Table 3). For ScDHOase, many H bonds with close distances were found. These bonds (<3 Å) included E184(A)–K188(C), K244(A)–E191(C), E184(B)–K188(D), K188(B)–E184(D), and N237(B)–K188(D). However, these interactions were not found in the crystallographically related dimer–dimer interface of EcDHOase (Table 3 and Figure 6H). The distance of these corresponding residues in EcDHOase was too far to interact with each other. In addition, the loop in EcDHOase corresponding to the tetramerization loop of ScDHOase was too short to interconnect (Figure 6I). Compared with BaDHOase, EcDHOase, and huDHOase, ScDHOase had the longest loop 7 among these DHOases (Figure 3F). These crucial residues at the tetramerization helices 1 and 2 and the loop for tetramerization in ScDHOase were not conserved and might be the reason why BaDHOase, EcDHOase, and huDHOase could not form a tetramer (Figure 3F).

The dimer–dimer interactions of ScDHOase were dependent on pH (Table 3). Many H bonds were formed at the dimer–dimer interface, but only three H bonds, namely, K188(A)–E184(C), K188(B)–E184(D), and K240(B)–E191(D), were persistently involved in the tetramerization of ScDHOase regardless of pH conditions. Several H bonds, such as K188(A)–N237(C), N196(A)–N295(C), E184(B)–K188(D), K188(B)–N237(D), E191(B)–K240(D), and N295(B)–K198(D), formed at pH 6.0, 6.5, and 7.0 but not at pH 9.0. For comparison, 16, 15, 11, and 9 H bonds were formed at the dimer–dimer interface of ScDHOase at pH 7, 6.5, 6, and 9, respectively (Table 3). Considering the amount of the H bonds formed, the tetramer stability of ScDHOase might follow the order: pH 7.0 > pH 6.5 > pH 6.0 > pH 9.0.

### 3.7. Dimerization Mode of ScDHOase

**Figure 7:**
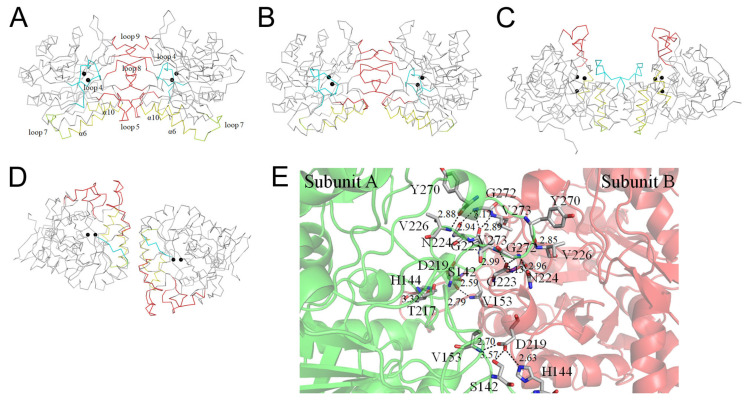
Dimerization mechanism. (**A**) The Cα trace for showing the dimerization pattern of ScDHOase. (**B**) The dimerization pattern of EcDHOase. (**C**) The dimerization pattern of huDHOase. (**D**) The dimerization pattern of BaDHOase. The dimerization patterns of EcDHOase, huDHOase, and BaDHOase differed significantly. Although the monomer–monomer contact pattern of ScDHOase roughly looked like that of EcDHOase, the interactive residues were almost different. Only the H bond of G272–G223 in ScDHOase could be found at the monomer–monomer interface of EcDHOase. (**E**) The formation of hydrogen bonds at the monomer–monomer interface of ScDHOase. Although the monomer–monomer contact pattern of ScDHOase roughly looks like that of EcDHOase, the interactive residues are almost different. Only a hydrogen bond of G272–G223 in ScDHOase could be found at the monomer–monomer interface of EcDHOase.

We analyzed and compared the monomer–monomer interface of ScDHOase (Figure 7A), EcDHOase (Figure 7B), huDHOase (Figure 7C), and BaDHOase (Figure 7D) to assess whether their dimer formation mechanisms were also different. The dimerization patterns of EcDHOase [16], huDHOase [15], and BaDHOase [18] differed significantly. Given the same type of enzyme, we focused on the comparison of ScDHOase and EcDHOase. The loops 5, 8, and 9 were involved in the dimer AB formation of ScDHOase (Figure 7A). The length of the loop 5 in ScDHOase was 6-amino acid residues longer than that in EcDHOase (Figure 3F and Figure 7B). The ScDHOase monomers A and B were interconnected through many H bonds (Table 4 and Figure 7E) and hydrophobic interactions. Although the monomer–monomer contact pattern of ScDHOase roughly looked like that of EcDHOase (Figure 7A,B), the interactive residues were almost different (Figure 3F). Only the H bond of G272–G223 in ScDHOase could be found at the monomer–monomer interface of EcDHOase. The monomer–monomer interface of ScDHOase was further stabilized using the hydrophobic core, namely, P150 (loop 5), V153 (loop 5), L154 (loop 5), I218 (α9), W221 (α9), A222 (loop 8), P225 (loop 8), F228 (loop 8), A268 (loop 9), and V273 (loop 9). Only P225 of these hydrophobic interactions could be found in EcDHOase (Figure 3F). Given that these critical residues for the ScDHOase dimerization (Table 4 and Figure 7E) were not conserved in EcDHOase, we concluded that their dimer formation mechanisms are different.

### 3.8. Malate Binding Mode

**Figure 8:**
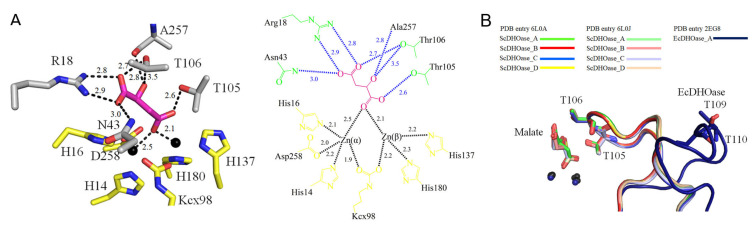
Malate binding mode. (**A**) The active site of subunit A with malate. Arg18, Asn43, Thr105, Thr106, and Ala257 were involved in malate binding. Residues required for metal binding are colored in yellow. (**B**) Superposition of the ScDHOase and EcDHOase complexes. ScDHOase bound malate via the loop-in mode for each subunit. As compared, EcDHOase bound non-substrate ligand via the loop-out mode; that is, the loop (dark blue) did not interact with the ligand or with the rest of the active site of EcDHOase.

Previously, we have found that crystal structures of ScDHOase determined at pH 6.0, 6.5, 7.0, and 9.0 revealed only slight conformational changes at the active sites [43]. In this study, we solved the crystal structure of ScDHOase at pH 7.5 (PDB entry 6L0J). At pH 7.5, two metal ions, Arg18, Asn43, Thr105, Thr106, and Ala257 were involved in malate binding (Figure 8A). This binding mode of ScDHOase at pH 7.5 was similar to those at different pH values. ScDHOase bound malate via the loop-in mode (Figure 8B), i.e., Thr105 and Thr106 in the catalytic loop, were involved in malate binding. In comparison, EcDHOase bound non-substrate ligand via the loop-out mode; that is, the loop (dark blue) did not interact with the ligand or with the rest of the active site of EcDHOase [56]. To date, we have not found the loop-out mode of ScDHOase to bind ligand. Whether ScDHOase can bind ligand via the loop-out conformation is still unknown. Given that the flexible loop in ScDHOase is the longest among these DHOases (Figure 3F), they may be somehow different in their binding mechanisms.

### 3.9. Different Tetramerization Mechanisms of ScDHOase, DHPase, and ALLase

The oligomerization is a common property of proteins. More than 35% of all proteins are oligomeric in biological systems [57,58]. Protein complexes are under the evolutionary selection to assemble via ordered pathways [55]. Some proteins are in dynamic oligomerization equilibria between several states with distinct activities [55], such as the cases with the acetylcholine receptor [59] and DnaT replication restart protein [52,60]. Only when forming as a pentamer, the acetylcholine receptor and DnaT are active for catalysis and ssDNA binding, respectively. In this study, we found that ScDHOase could form dimers and tetramers. Except pseudomonal DHPases forming a dimer [23,24], other cyclic amidohydrolase members [21], such as DHPases and ALLases, are all tetramers. Although the ScDHOase, DHPase, and ALLase tetramers consisted of four classic (β/α)_8_-barrel structures, their oligomerization patterns and the contact residues were quite different (Figure 6). Despite the similarities, the structural comparison clearly demonstrated that the oligomerization mechanisms of these cyclic amidohydrolases differed.

The subunit-interacting residues of ScDHOase for the dimerization and the tetramerization were significantly different from those of huDHOase, BaDHOase, and EcDHOase (Figure 3, Figure 5, Figure 6 and Figure 7). The kinds of evolutionary selection to force these DHOases to self-assemble into different oligomer forms remain unknown. Although the de novo synthesis process of pyrimidines was conserved among all species, the gene products responsible for these activities differed (Figure 1). One line of structural evidence indicated that these DHOases might have different situations for oligomer evolution. The difference among these DHOases was that ScDHOase did not require the formation of multicomplexes with CPSase and ATCase. In fungi, CPSase and ATCase were covalently contained within a CAD-like polypeptide. Similar but different to human CAD, this CAD-like polypeptide contained a DHOase-like domain but lacked the catalytic activity. The fungi DHOase, as an example of ScDHOase in this study, was speculated to be no longer needed to coevolve with the CPSase and the ATCase gradually, which may explain why the residues for tetramerization in ScDHOase were not necessarily conserved among DHOases (Figure 3F).

### 3.10. Binuclear Metal Center within a Carbamylated Lysine

From a biochemical point of view, DHOase, DHPase, and ALLase belong to the cyclic amidohydrolase family [21,61,62] and catalyze various hydrolytic reactions at the cyclic amide ring. Almost all cyclic amidohydrolases had a cluster of four His, an Asp, and a carbamylated Lys to bridge the two metal ions. However, different metal contents were observed for AaDHOase and huDHOase. Only one Zn^2+^ ion is seen in the active site of each AaDHOase subunit, and the carbamylated Lys is replaced by an Asp [38,63]. However, three Zn ions of huDHOase are revealed using structural analysis [15]. The third Zn^2+^ ion in huDHOase, which had not been found in any DHOase, was functionally important for the catalysis of huDHOase. The third metal-binding site was not found in ScDHOase (Figure 3). However, the third metal-binding site of ScDHOase can be created by a single mutation (ScDHOase-T208E) [44]. The catalytic activity of ScDHOase-T208E was enhanced, compared with that of the wild-type di-metal enzyme. During evolution, the need for a higher DHOase stability and activity may drive the creation of a third metal ion binding site in huDHOase, which can be achieved by mutating a highly conserved position T in type II dihydroorotases to E, similar to huDHOase.

### 3.11. Dynamic Loop as a Part of the Catalytic Cycle in DHOase and DHPase

Based on the crystal structures of HYDase [64], DHPase [27], DHOase [16,65], and ALLase [30], the chemical mechanism of the binuclear metal center-containing cyclic amidohydrolases likely has three main steps [21]. (I) The hydrolytic water molecule must be activated for nucleophilic attack. (II) The amide bond of the substrate must be made electrophilic by the polarization of the carbonyl O bond. (III) The leaving group N must be protonated as the C–N bond is cleaved. The flexible loop in EcDHOase was also crucial for catalysis, supporting that the movement of this loop was part of the catalytic cycle [17]. However, this loop was not conserved among DHOases (Figure 3F) [41] and exhibited species-dependent selectivity for catalysis [19]. The complexed crystal structures of EcDHOase [17] or huDHOase [15] with DHO or the product-like inhibitor 5-fluoroorotate indicated the loop-out mode. However, our malate-complexed structures of ScDHOase indicated the loop-in mode (Figure 8). Structural evidence indicated that the two Thr residues (Thr105 and Thr106 in ScDHOase) important for stabilizing the transition state were involved in binding of malate, suggesting that the conclusion reached with EcDHOase was species-dependent. By using standard assay, malate (200 μM) could decrease the activity of DHOase by 6% [42]. Regardless of their different sequences and the binding modes, the flexible loop in DHPase [21,66] and DHOases [19], including ScDHOase (Figure 8), was crucial for the catalysis. Thus, the dynamic loop in DHOase and DHPase should be suitable drug targets for inhibiting the pyrimidine metabolism selectively [20,66]. We propose that a compound that could partially occupy the active site and stably lock off the loop movement of DHOase would be a good candidate as a drug lead for developing potent inhibitor toward DHOase, and this speculation should be elucidated biochemically and structurally.

## 4. Conclusions

In this study, we identified that ScDHOase could be dimers and tetramers both in the crystalline state and in solution. The structures of ScDHOase, huDHOase, EcDHOase, and BaDHOase were compared. The subunit-interacting residues of ScDHOase for dimerization and tetramerization are significantly different and this finding might be due to the evolutionary diversity.

## Figures and Tables

**Table 1:** Data collection and refinement statistics.

Data Collection		
Crystal	Malate/pH 7	Malate/pH 7.5
Source	SPring8-BL44XU	NSRRC-15A
Wavelength (Å)	0.9 Å	1.0 Å
Resolution (Å)	40.82–1.79	28.07–1.93
Space group	P2_1_	P2_1_
Cell dimension *a*, *b*, *c* (Å)/*β* (°)	85.57, 88.54, 103.08/95.73	85.55, 88.33, 103.20/95.60
Redundancy	4.1 (4.1)	4.1 (4.0)
Completeness (%)	99.45 (97.94)	99.90 (99.90)
<I/σI>	24.7 (4.14)	18.17 (1.16)
CC_1/2_	0.976 (0.907)	0.875 (0.480)
Refinement		
Resolution (Å)	40.82–1.79	28.07–1.93
No. reflections	143267	100525
*R*_work_/*R*_free_	0.178/0.207	0.183/0.229
No. atoms		
ligands	44	44
Zinc	8	8
Water	1643	1212
Protein residues	1454	1454
r.m.s deviations		
Bond lengths (Å)	0.008	0.009
Bond angles (°)	1.23	1.27
Ramachandran plot		
Favored (%)	96.37	95.61
Allowed (%)	3.42	3.35
Outliers (%)	0.21	1.05
PDB entry	6L0A	6L0J

Values in parentheses are for the highest resolution shell. CC_1/2_ is the percentage of correlation between intensities of random half-data sets.

**Table 2:** Thermostability of ScDHOase.

Temperature	The Decreased Activity (%)	
	ScDHOase	huDHOase
40 °C	3	9
50 °C	21	48
60 °C	48	89
70 °C	91	100

Protein (1 μM) was incubated at temperatures ranging from 40 °C to 70 °C for 10 min.

**Table 3:** The formation of hydrogen bonds at the dimer AB–dimer CD interface of ScDHOase and the corresponding residues at the crystallographically related dimer AB–dimer A’B’ interface of EcDHOase.

ScDHOase	pH				EcDHOase	
Hydrogen Bonds	6.0	6.5	7.0	9.0	Corr. Residues	Dist. [Å]
E184(A)–K188(C)		3.40	2.53	2.83	K181(A)/D185(A’)	5.48
K188(A)–E184(C)	2.70	2.77	3.14	2.78	D185(A)/K181(A’)	5.84
K188(A)–N237(C)	2.93	2.95	3.42		D185(A)/I229(A’)	9.37
E191(A)–K244(C)			3.09		R188(A)/E236(A’)	4.08
K195(A)–Q294(C)		3.38			None(A)/M285(A’)	
N196(A)–N295(C)	3.44	3.41	3.84		None(A)/N286(A’)	
N237(A)–K188(C)		3.47	3.02	2.90	I229(A)/D185(A’)	7.64
K240(A)–D192(C)			3.46		Q232(A)/D189(A’)	8.26
K244(A)–E191(C)			2.86	3.82	E236(A)/R188(A’)	4.64
N295(A)–N196(C)		3.57			N286(A)/None(A’)	
E184(B)–K188(D)	2.59	2.81	2.83		K181(B)/D185(B’)	5.12
K188(B)–E184(D)	2.62	2.83	2.64	3.64	D185(B)/K181(B’)	4.79
K188(B)–N237(D)	2.88	2.69	3.11		D185(B)/I229(B’)	7.94
E191(B)–K240(D)	3.59	3.68	3.60		R188(B)/E236(B’)	6.05
D192(B)–K240(D)				3.09	D189(B)/E236(B’)	4.47
N237(B)–K188(D)		3.13	2.83	3.71	I229(B)/D185(B’)	9.34
K240(B)–K188(D)	3.81				Q232(B)/D185(B’)	10.61
K240(B)–E191(D)	3.69	3.76	3.79	3.64	Q232(B)/R188(B’)	9.37
K240(B)–D192(D)	3.01	3.10			Q232(B)/D189(B’)	7.21
N295(B)–N196(D)				3.48	N286(B)/None(B’)	
N295(B)–K198(D)	3.55	3.47	3.86		N286(B)/None(B’)	
N295(B)–K195(D)			3.62		N286(B)/None(B’)	

The formation of hydrogen bonds at the dimer–dimer interface of ScDHOase was analyzed by using PISA (Protein Interfaces, Surfaces, and Assemblies), which is an automatic analytical tool for macromolecular assemblies in the crystalline state.

**Table 4:** The formation of hydrogen bonds at the monomer–monomer interface of ScDHOase.

Subunit A	Subunit B	Dist. [Å]
S142 [OG]	D219 [OD2]	2.63
H144 [NE2]	D219 [OD2]	3.57
V153 [N]	D219 [OD1]	2.70
T217 [OG1]	H144 [NE2]	3.32
D219 [OD1]	V153 [N]	2.79
D219 [OD2]	S142 [OG]	2.59
G223 [O]	G272 [N]	3.17
G223 [O]	V273 [N]	2.89
N224 [OD1]	G272 [N]	2.94
V226 [N]	Y270 [O]	2.88
Y270 [O]	V226 [N]	2.85
G272 [N]	G223 [O]	3.13
G272 [N]	N224 [OD1]	2.96
V273 [N]	G223 [O]	2.99

The formation of hydrogen bonds and salt bridges at the monomer–monomer interface of ScDHOase was analyzed by using PISA (Protein Interfaces, Surfaces, and Assemblies), which is an automatic analytical tool for macromolecular assemblies in the crystalline state.

## Data Availability

Atomic coordinates and related structure factors were deposited in the PDB with accession codes 6L0A and 6L0J.

## Sample Availability

Not available.
